# Silent cerebral lesions after catheter ablation for atrial fibrillation using cryoballoon, hotballoon, laserballoon and radiofrequency catheters: a Bayesian network meta-analysis

**DOI:** 10.3389/fcvm.2024.1510468

**Published:** 2025-01-14

**Authors:** Tiantian Zheng, Youjin Kong, Li Wu, Chenxia Wu, Wei Mao, Xinbin Zhou

**Affiliations:** ^1^ Department of Cardiology, The First Affiliated Hospital of Zhejiang Chinese Medical University (Zhejiang Provincial Hospital of Chinese Medicine), Hangzhou, Zhejiang, China; ^2^ Department of Cardiology, Affiliated Zhejiang Hospital, Zhejiang University School of Medicine, Hangzhou, China; ^3^ Zhejiang Key Laboratory of Integrative Chinese and Western Medicine for Diagnosis and Treatment of Circulatory Diseases, Hangzhou, China

**Keywords:** atrial fibrillation, silent cerebral lesions, cryoballoon ablation, hotballoon ablation, laserballoon ablation, radiofrequency ablation, network meta-analysis

## Abstract

**Background:**

Catheter ablation (CA) is an effective therapeutic option for patients with symptomatic atrial fibrillation (AF). Previous studies have reported silent cerebral lesions (SCLs) detected by magnetic resonance imaging (MRI) after different CA techniques; however, the results were controversial. Therefore, we performed this network meta-analysis (NMA) to assess the incidence of SCLs after cryoballoon, hotballoon, laserballoon, and radiofrequency ablation (RFA).

**Methods:**

Databases such as PubMed, Embase, and the Cochrane Library were searched systematically. Both pairwise meta-analysis (PMA) and NMA were conducted. The primary outcome was the incidence of new SCLs on MRI after CA procedures.

**Results:**

Nine studies were analyzed and 1,057 patients were enrolled. Laserballoon ablation (LBA) had a higher incidence of SCLs than cryoballoon ablation (CBA) [odds ratio [OR] = 1.86, 95% confidence interval [CI] 1.06–3.27, *p* = 0.032] in the PMA, while no significant difference was detected between the CA techniques according to the NMA. The surface under the cumulative ranking curve (SUCRA) values indicated that CBA may be the best therapeutic option (SUCRA = 81.1%). The NMA results demonstrated similar procedure-related complication rates and mean activated clotting time between CBA (SUCRA = 53.7%, 66.3%), hotballoon ablation (HBA) (SUCRA = 81.5%, 43.6%), LBA (SUCRA = 3.39%, 42.8%) and RFA (SUCRA = 61.3%, 47.3%). LBA therapy required significantly more procedure time than CBA [weighted mean difference (WMD) = 24.36 min, 95% CI 12.51–36.21 min, *p* = 0.00].

**Conclusions:**

CBA treatment had lower incidence of post-procedural SCLs and took less procedure time compared with LBA for patients with AF. The procedure-related complications were comparable between CBA, LBA, HBA and RFA.

**Systematic Review Registration:**

PROSPERO, identifier (CRD42024511110).

## 1 Introduction

Atrial fibrillation (AF) is a common cardiac arrhythmia in clinical practice, and can lead to increased risk of cognitive decline and ischemic stroke (1, 2). Many guidelines have recommended catheter ablation (CA) as first-line treatment for the purpose of restoring and maintaining sinus rhythm in patients with symptomatic AF (3). Several previous studies have evaluated neurocognitive outcomes and reported the incidence of silent cerebral lesions (SCLs) detected by cerebral magnetic resonance imaging (MRI) following CA for AF, ranging from 1.7 to 38% (4, 5). SCLs, also known as asymptomatic cerebral lesions, are defined as cerebral lesions that display radiological evidence of focal ischemia without resulting in acute symptoms (6, 7). SCLs were reported to be associated with progressive neurocognitive decline and an increased risk of developing dementia (8). Different ablation techniques for AF have been compared with regard to the rates of SCLs; However, the results are controversial and most of the studies included small patient numbers (6). We conducted the present systematic review and network meta-analysis (NMA) to fully assess and compare the incidence and characteristics of SCLs following cryoballoon ablation (CBA), laser balloon ablation (LBA), hot balloon ablation (HBA) and irrigated radiofrequency ablation (RFA) therapy for patients with AF.

## 2 Materials and methods

### 2.1 Literature search strategy and study selection

We searched scientific databases and websites, such as PubMed (MEDLINE), Embase, the Cochrane Library, and ClinicalTrials.gov up to June 2024. The following keywords and their variants were used: “atrial fibrillation”, “catheter ablation” and “silent cerebral lesions”. The reference lists of the relevant articles were further browsed. Clinical trials that met the following criteria were included: (1) article in English, (2) original data comparing cryoballoon, hotballoon, laserballoon and irrigated radiofrequency catheter ablation for AF, (3) cerebral MRI examination was performed after CA treatment and (4) adequate data regarding the outcomes of interest.

### 2.2 Data collection and quality assessment

Two authors (TTZ and YJK) extracted the data of interest and assessed the qualities independently. The Cochrane Collaboration tool was used to assess the methodological qualities of randomized controlled trials (RCTs) (9), while the ROBINS-I tool (10) was applied for the non-randomized trials. Any disagreement was resolved by discussion or by referral to a third assessor where necessary. The following data from the included trials were extracted: the features of the studies, the baseline characteristics of the participants, CA strategies, and outcomes of interest.

### 2.3 Primary and secondary outcomes

The primary outcome was the occurrence of new SCLs on MRI examination following CBA, HBA, LBA, and RFA treatments. Secondary outcomes of interest included total procedure-related complications, CA procedure time and mean activated clotting time (ACT) during the procedure.

### 2.4 Statistical analysis

Categorical variables were represented as percentages, while continuous variables were expressed as mean ± standard deviation (SD). Data analysis for pairwise meta-analysis (PMA) was conducted using the STATA software (v15.1). The estimated weighted mean difference (WMD) and odds ratio (OR) with their 95% confidence interval (CI) were calculated. For Bayesian network meta-analysis, the R software (version 3.6.2) was applied to calculate the mean difference (MD)/OR and the 95% credible interval (CrI). We performed Markov chain Monte Carlo algorithm for sampling the posterior probabilities from 100,000 iterations via Gibbs sampling. We used the surface under the cumulative ranking curve (SUCRA) probabilities to rank each CA therapy type for certain outcomes. Pairwise heterogeneity was evaluated with the chi-squared test. If moderate to significant heterogeneity was observed, additional subgroup and sensitivity analyses were performed. The node-splitting method was used to test the consistency (11). Publication bias was evaluated by funnel plots. Egger's and Begg's tests were also applied to statistically assess the bias. The protocol of this network meta-analysis was registered in the PROSPERO (CRD42024511110).

## 3 Results

### 3.1 Eligible studies and characteristics

**Figure 1:**
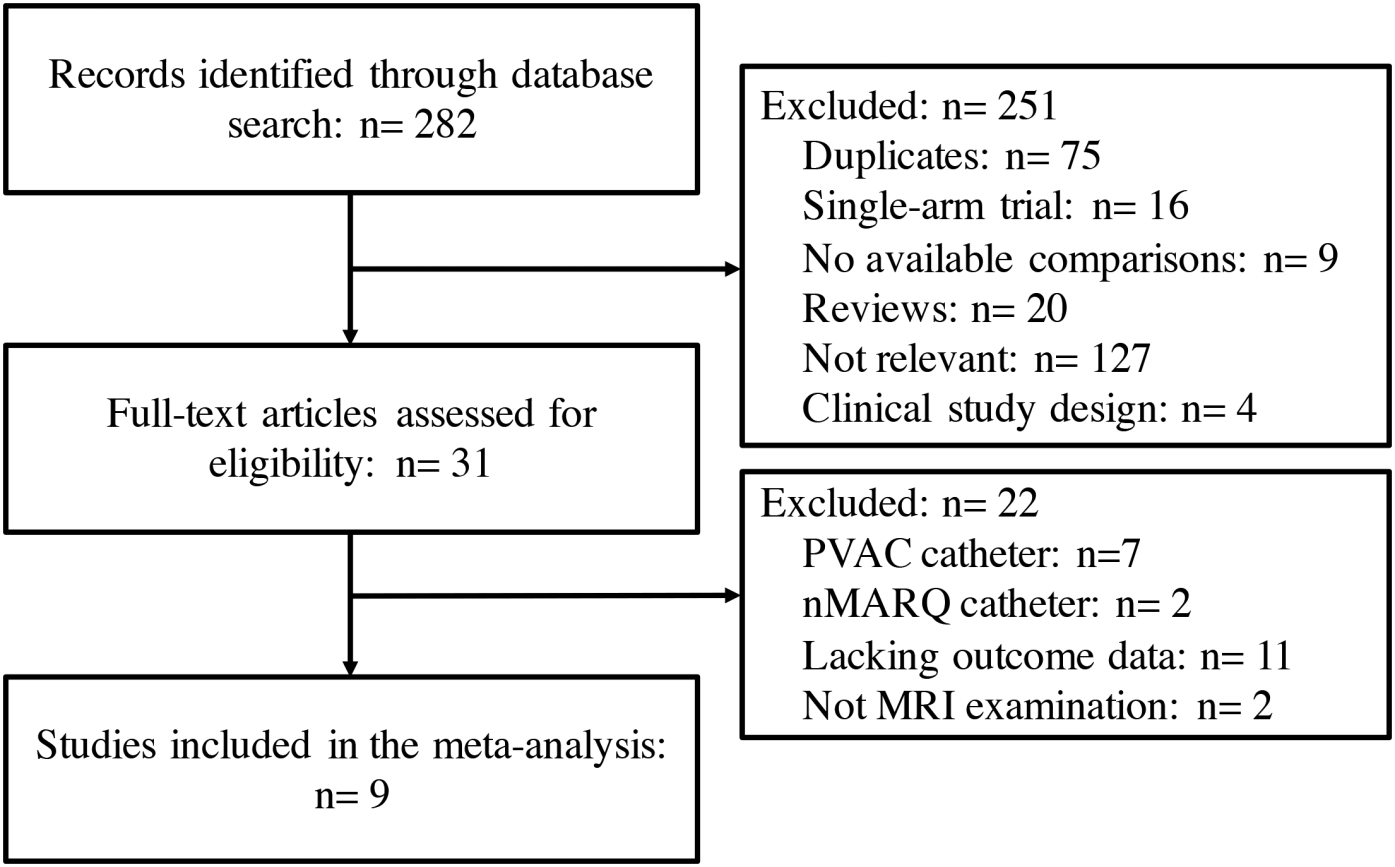
Flow chart of the systematic literature research.

**Figure 2:**
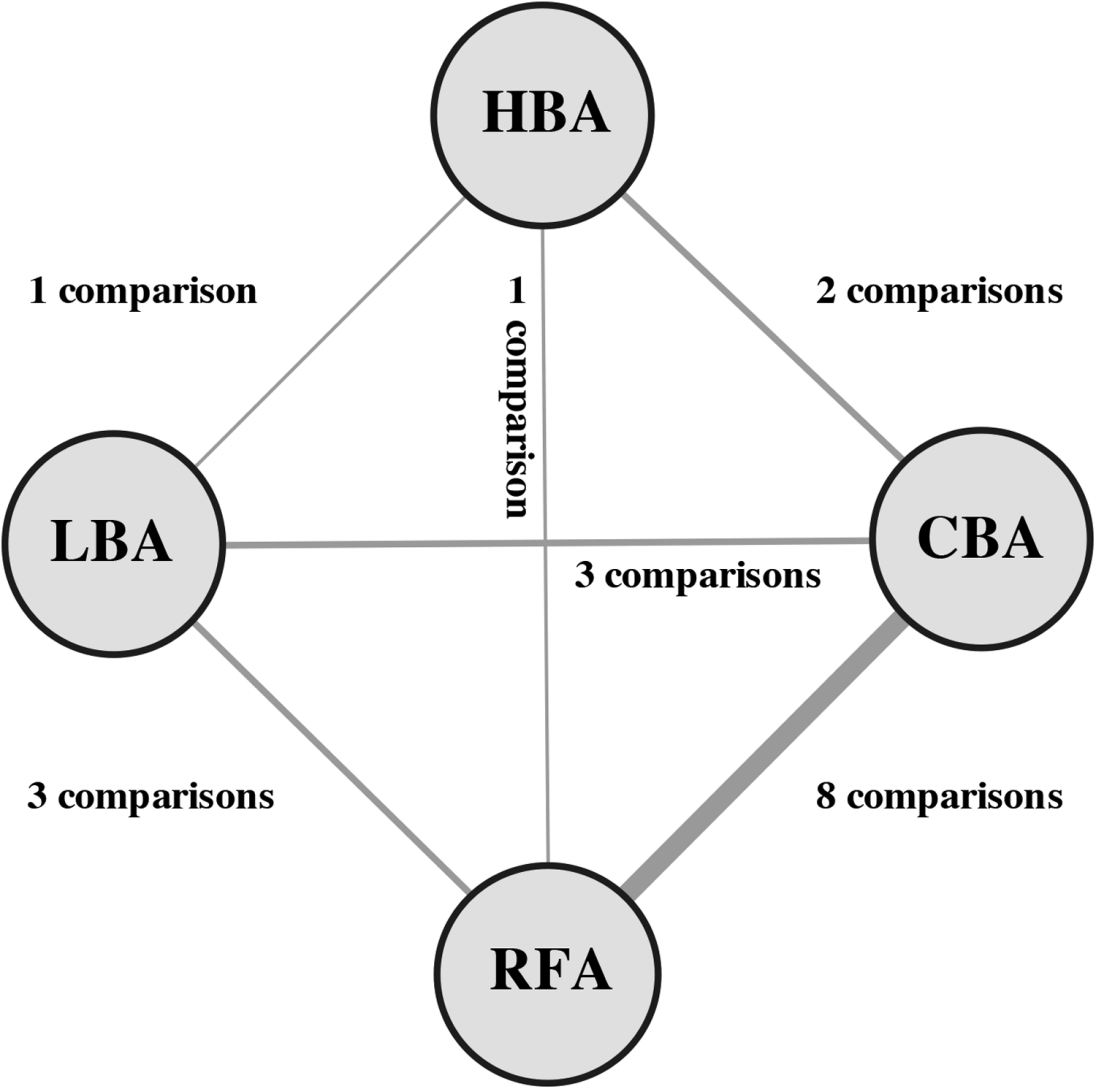
Network of comparisons included in the analyses. Evidence structure of direct comparisons included for network meta-analysis. The thickness of the line corresponds to the number of comparisons. HBA, hot balloon ablation; LBA, laser balloon ablation; CBA, cryoballoon ablation; RFA, radiofrequency ablation.

A total of 282 clinical trials were identified after electronic database searching, and 9 studies (12–20) with a total of 1,057 participants were eventually enrolled. (Figure 1) The features and baseline characteristics of these studies and participants are exhibited in Table 1. Briefly, across the trials, one study compared HBA with CBA (15), five trials compared CBA with RFA (12–14, 16, 19), two studies compared LBA, CBA and RFA (17, 20), and one study compared these four CA therapies concurrently (18). (Figure 2) Three studies only included patients with paroxysmal AF (PAF) (12, 17, 18), while the remaining studies enrolled mixed cohort of patients with AF, including both persistent AF (PerAF) and PAF patients. The CBA group contained 419 patients, the HBA group had 108 patients, the LBA group contained 128 patients, and the RFA group had 402 patients. The mean age across studies was 61.0 years, and the median CHA2DS2-VASc score was 1.8. Oral anticoagulant (OAC) treatments during the procedure were uninterrupted in four studies (13, 15, 18, 19), while discontinued in four studies (12, 14, 16, 17). According to the quality assessment results via the Cochrane Collaboration tool (9) and ROBINS-I tool (10), all the studies included in the analysis were of relatively good qualities. There was no significant publication bias according to the results of funnel plot (Supplementary Figure S1) and Egger's and Begg's tests (Egger's: *p* = 0.395; Begg's: *p* = 0.343), based on the primary outcome.

**Table 1:** Baseline characteristics of the included studies.

Study	Year	Study type	*N*	Treatment	PAF (%)	Mean age (years)	Male (%)	Mean LVEF (%)	Mean LAd (mm)	DM (%)	Hypertention (%)	Stroke/TIA (%)	Median CHA2DS2-VASc	OAC during the procedure
Nakamura	2019	Prospective	123	HBA vs. CBA	95.1	65	68.3	63	38	17.1	46.3	6.5	1.9	Uninterrupted
Glowniak	2019	Prospective	48	CBA vs. RFA	89.6	59	75	64.9	42.8	20.8	75	8.3	1.7	Uninterrupted
von Bary	2015	Prospective	28	CBA vs. RFA	73.1	63	NR	58	43	NR	61.5	NR	NR	Uninterrupted
Herrera Siklody	2011	Prospective	50	CBA vs. RFA	56	61	70	NR	41.1	NR	60	NR	1.7	Discontinued
Gaita	2011	Prospective	72	CBA vs. RFA	100	56	68.1	63.5	42	NR	54.2	NR	NR	Discontinued
Neumann	2011	Prospective	89	CBA vs. RFA	80.9	57	62.9	60	52	4.5	55.1	NR	NR	Discontinued
Wissner	2014	Prospective	86	LBA vs. CBA vs. RFA	77	63	63	64	42	9	66	8	2	NR
Schmidt	2013	RCT	99	LBA vs. CBA vs. RFA	100	65	NR	59	40	6	73	7	2	Discontinued
Tokuda	2022	Prospective	462	HBA vs. LBA vs. CBA vs. RFA	100	59.7	79.4	64.4	36.9	7.8	41.1	4.3	1.3	Uninterrupted

PAF, paroxysmal atrial fibrillation, LVEF, left ventricular ejection fraction, LAd, left atrial diameter, DM, diabetes mellitus, TIA, transient ischemia attack, OAC, oral anticoagulant, NR, not reported, HBA, hot balloon ablation, LBA, laser balloon ablation, CBA, cryoballoon ablation, RFA, radiofrequency ablation, RCT, randomized controlled trial.

### 3.2 Primary endpoint

#### 3.2.1 SCLs occurrence

**Figure 3:**
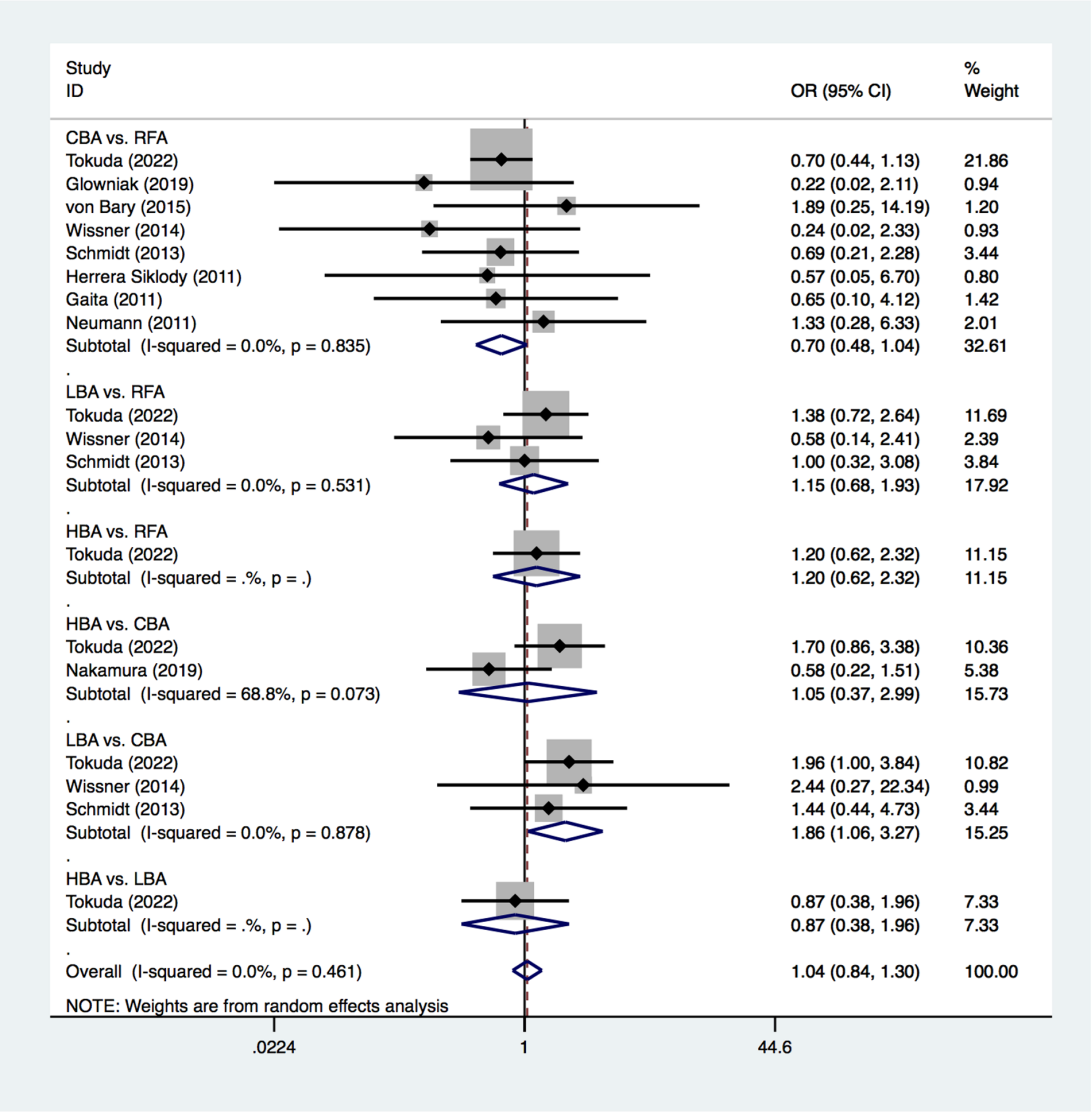
Forest plot for the outcome of SCLs from pairwise meta-analysis. HBA, hot balloon ablation; LBA, laser balloon ablation; CBA, cryoballoon ablation; RFA, radiofrequency ablation; OR, odds ratio.

All of the included studies reported on the outcome of new SCLs on MRI after CA treatments for AF. The PMA analysis exhibited that, LBA had significantly higher SCL occurrence than CBA after AF procedure (25.0% vs. 20.8%, OR = 1.86, *p* = 0.032) (Figure 3). No significant heterogeneity was detected (I^2^ = 0%). No significant differences were found regarding to SCL occurrence between CBA vs. RFA (OR = 0.70, *p* = 0.075), LBA vs. RFA (OR = 1.15, *p* = 0.605), HBA vs. RFA (OR = 1.20, *p* = 0.590), HBA vs. CBA (OR = 1.05, *p* = 0.926), and HBA vs. LBA (OR = 0.87, *p* = 0.733) (Figure 3).

**Figure 4:**
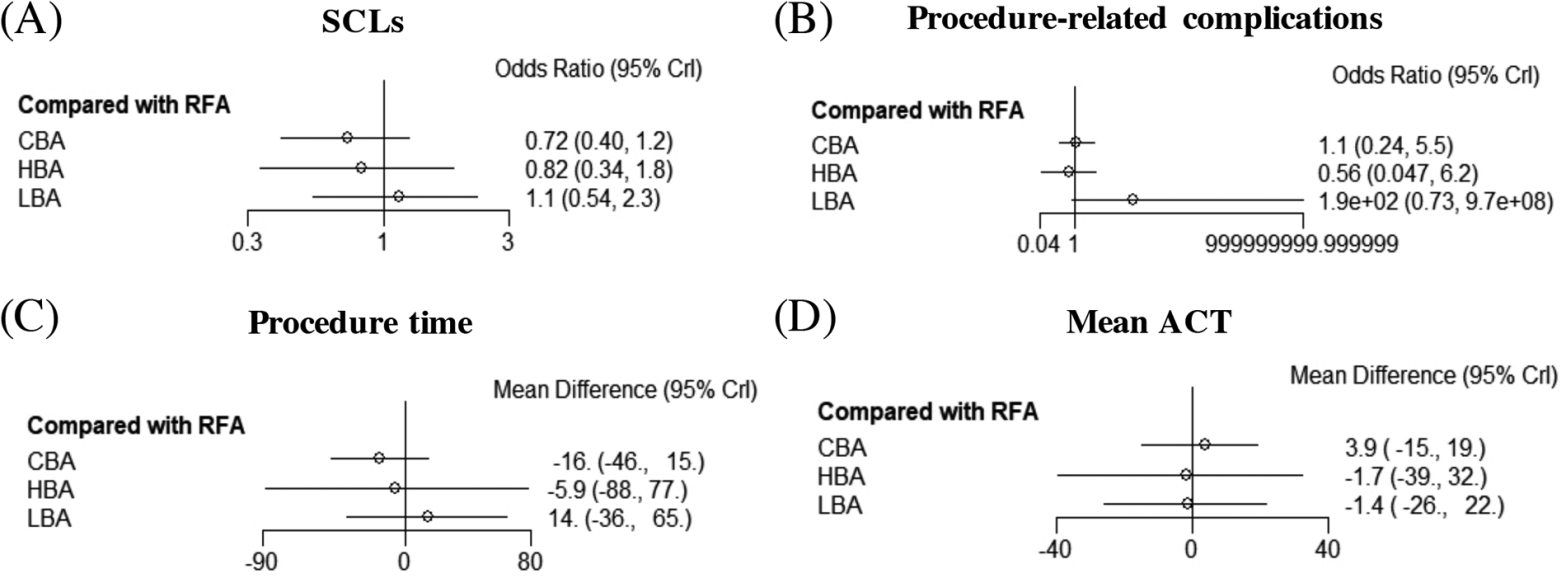
Forest plots of network meta-analysis for the primary and secondary outcomes. **(A)** SCLs, **(B)** procedure-related complications, **(C)** procedure time, **(D)** mean ACT. SCLs, silent cerebral lesions; ACT, activated clotting time; HBA, hot balloon ablation; LBA, laser balloon ablation; CBA, cryoballoon ablation; RFA, radiofrequency ablation.

**Figure 5:**
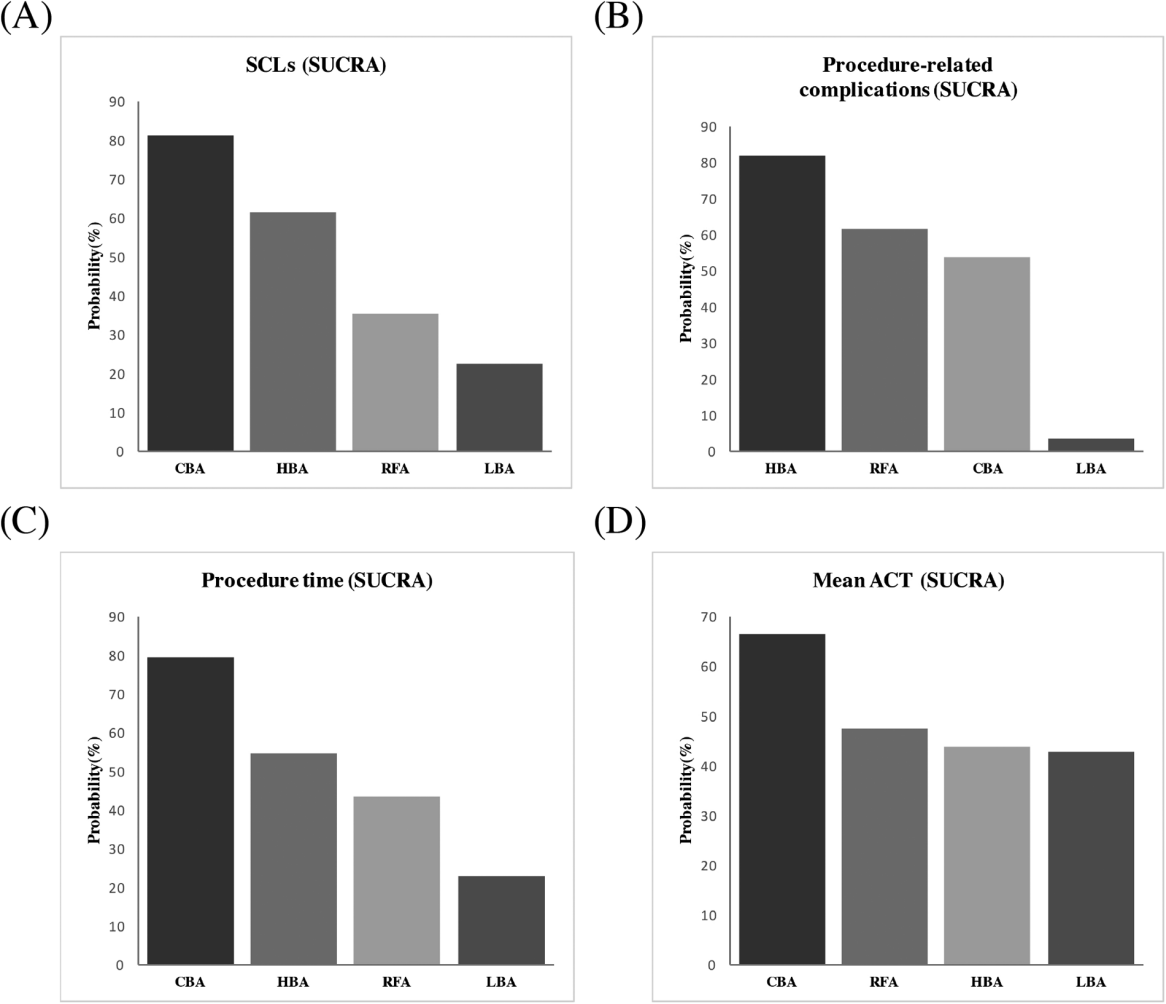
Ranking probabilities according to SUCRA. **(A)** SCLs, **(B)** procedure-related complications, **(C)** procedure time, **(D)** mean ACT. SUCRA, surface under the cumulative ranking curve; SCLs, silent cerebral lesions; ACT, activated clotting time; HBA, hot balloon ablation; LBA, laser balloon ablation; CBA, cryoballoon ablation; RFA, radiofrequency ablation.

Results from the NMA demonstrated that, compared with RFA, CBA (OR = 0.72), HBA (OR = 0.82) and LBA (OR = 1.1) showed similar occurrence rates of SCLs after AF procedure. (Figure 4) No significant differences in SCLs recurrence was found between these four CA treatments for AF (Supplementary Table S1). The SUCRA results indicated that, CBA may be the optimal therapeutic option (SUCRA = 81.1%), followed by HBA (SUCRA = 61.4%), RFA (SUCRA = 35.1%) and LBA (SUCRA = 22.4%) (Figure 5). Good consistency was detected, as the deviance information criterion (DIC) values were similar regarding to the primary endpoint (DIC 33.73, I^2^ = 0% vs. DIC 33.70, I^2^ = 0%).

#### 3.2.2 Total SCLs numbers on post-procedure MRI

Four studies (12, 16, 17, 20) provided data on the total SCLs numbers on post-procedure MRI between different CA strategies. PMA demonstrated similar SCL numbers between CBA vs. RFA (OR = 0.67, *p* = 0.075) and LBA vs. CBA (OR = 2.16, *p* = 0.143). LBA treatment was found to have less total SCL numbers than RFA (OR = 0.46, *p* = 0.046). (Supplementary Figure S2). We found significant heterogeneity in the comparison between CBA and RFA (I^2^ = 74.3%), while this was not detected for the comparisons between LBA vs. RFA (I^2^ = 0%) and LBA vs. CBA (I^2^ = 0%). NMA was not conducted due to the lack of sufficient comparisons.

### 3.3 Secondary endpoints

#### 3.3.1 Procedure-related complications

Five of the included trials (13–15, 17, 20) provided data on procedure-related complications. According to the results of the PMA, the CBA (6.6%), HBA (5.1%), LBA (2.6%) and RFA groups (4.9%) had comparable complication rates (CBA vs. RFA: OR = 1.09, *p* = 0.896; LBA vs. RFA: OR = 2.65, *p* = 0.535; HBA vs. CBA: OR = 0.54, *p* = 0.394; LBA vs. CBA: OR = 2.41, *p* = 0.576). No significant heterogeneities were found for these comparisons (Supplementary Figure S3).

The NMA analysis demonstrated comparable results regarding to the procedure-related complications between CBA (SUCRA = 53.7%), HBA (SUCRA = 81.5%), LBA (SUCRA = 3.39%) and RFA groups (SUCRA = 61.3%) (Figures 4, 5). Good consistency was detected with similar DIC values. (DIC 13.95, I^2^ = 0% vs. DIC 13.97, I^2^ = 0%).

#### 3.3.2 Procedure time

Eight studies (12–17, 19, 20) reported comparison data concerning procedure time. The results of the PMA indicated that, LBA required remarkably longer procedure time during CA compared to CBA (WMD = 24.36 min, *p* = 0.00). Similar procedure times were seen between CBA vs. RFA (WMD = −16.10 min, *p* = 0.237), LBA vs. RFA (WMD = 18.18 min, *p* = 0.537) and HBA vs. CBA (WMD = 10.00 min, *p* = 0.061). The moderate to significant heterogeneities for these comparisons should be noted (I^2^ = 93.9%, 90.4%, and 94.7%, respectively). (Supplementary Figure S4).

The results of NMA exhibited that, CBA (WMD = −16. 0 min, SUCRA = 79.1%), HBA (WMD = −5.9 min, SUCRA = 54.6%) and LBA therapies (WMD = 14.0, SUCRA = 22.9%) had comparable procedure time, compared with RFA therapy (SUCRA = 43.4%). There was no significant difference regarding to the procedure time between the four CA treatments (Figures 4, 5).

#### 3.3.3 Mean ACT during procedure

Five studies (12, 15–17, 20) reported on the mean ACT during the procedure. The PMA demonstrated comparable mean ACT during CA for AF between CBA vs. RFA (WMD = 1.3 s, *p* = 0.892), LBA vs. RFA (WMD = −10.75 s, *p* = 0.257), HBA vs. CBA (WMD = −6.0 s, *p* = 0.542) and LBA vs. CBA (WMD = 1.15 s, *p* = 0.917) (Supplementary Figure S5). The NMA results also showed no significant differences regarding to the mean ACT during the procedure between CBA (SUCRA = 66.3%), HBA (SUCRA = 43.6%), LBA (SUCRA = 42.8%), and RFA (SUCRA = 47.3%) (Figures 4, 5).

## 4 Discussion

To our knowledge, this is the first network meta-analysis that comprehensively compared the incidence of SCLs after CBA, HBA, LBA and RFA treatments for AF. The main findings were: (1) CBA therapy was associated with a significantly lower incidence of SCLs compared with LBA for patients with AF in the PMA, (2) no significant difference was detected regarding the incidence of SCLs between CBA, HBA, LBA and RFA for AF in the NMA, (3) LBA had less total SCLs numbers on MRI than RFA, (4) the four CA techniques showed comparable procedure related complications, and (5) LBA treatment required longer procedure time compared with CBA.

CA has been the recommended therapy for symptomatic and drug-refectory patients with AF, to restore and maintain sinus rhythm (3); however, CA within the left atrium may lead to the formation of SCLs, which could be detected via MRI (6). While symptomatic embolism events, such as stroke and transient ischemic attack after CA for patients with AF are rare (21), increasing evidence suggests that the incidence of SCLs detected by cerebral MRI is relatively high, and has been reported to range from 1.7% to 42% (22, 23). Various factors could contribute to formation of SCLs during CA, such as air or thrombus, coagulum on the catheter, gas bubble formation, patient-related factors, electrical cardioversion during the procedure and certain types of ablation device (6, 18).

Though many previous studies have investigated the influence of these factors on the incidence of post-procedural SCLs, no congruent result has been reached. Several studies have also compared the occurrence of SCLs after different ablation techniques, whereas results are also controversial (6). The RFA therapy for AF has been widely applied, and in recent years, many balloon-based ablation techniques, such as CBA, HBA and LBA, have also been developed, which have demonstrated comparable clinical efficacy and safety compared with RFA (24–26). In the present study, CBA was found to have a significantly lower incidence of SCLs than LBA in the PMA, but not in the NMA; this was consistent with the previous prospective, multicenter studies that investigated the incidence of SCLs after CBA and LBA respectively (20). Balloon-based techniques have been introduced and emerged as valuable alternatives to the traditional RFA procedure (27). Previous histological studies have reported that conventional RFA resulted in more extensive endothelial cell destruction, which may lead to the release of endothelial inflammatory cytokines and the activation of platelets, and increase the risk of thrombus formation (28). However, CBA has a lower thrombogenic nature, due to its well-delineated, discrete lesions that preserve the tissue ultrastructure (29).

Compared with CBA, LBA has the advantage of direct endocardial visualization during the procedure (30); however, the residual blood between the balloon and left atrial wall during procedure may cause thrombus formation (18). In addition, the risk of SCLs after the procedure is associated with prolonged procedural times (31). In the present study, the procedure time was much longer with LBA than that with CBA. This was also consistent with the recent study by Wu et al., which demonstrated that LBA needed the most procedural time compared with CBA and HBA (27). LBA has the advantage of enabling a more precise titration of ablation lesions, whereas this would take up more procedure time, in contrast to the “single shot” techniques, such as HBA and CBA. Moreover, LBA lacks a specific mapping catheter within its system for the real-time recording of PV potential, which can further extend the procedural time, as another mapping catheter is required to verify PVI (27). These risk factors may in part explain the higher occurrence of SCLs found in the LBA treatment compared with CBA.

Although LBA had a higher incidence of SCLs than CBA, it comprised less of the total SCL numbers than RFA in the PMA. As mentioned above, RFA tends to have a higher risk of thrombus formation due to the extensive endothelial cell destruction. Substantially more frequent occurrence of SCLs was also reported when using duty-cycled phased pulmonary vein ablation catheter (PVAC) and the NMARQ multielectrode catheter (22, 32). The newly introduced technology, high-power, short-duration ablation (HPSD) was also shown to be associated with an increased risk of SCLs (33, 34).

However, notably, comparable occurrences and total numbers of SCLs were seen between the four techniques in the NMA, which used direct and indirect evidence. Possible explanations may be as follows: First, the total trial number and the studies included in the comparison between LBA and CBA were relatively small, which may inevitably cause bias. However, the studies included in the PMA for the outcome of SCLs between LBA vs. CBA and LBA vs. RFA were of good quality, and according to the GRADE approach (35), the results may still have a solid foundation of evidence. Second, the exact definitions of SCLs and the modalities of MRI scanning differed between studies, which may have affected the results. As it was reported that, a higher incidence of SCLs was seen with the technique of high-resolution diffusion-weighted imaging (DWI) (7). Third, not all the included studies assessed the incidence of SCLs before the CA procedure. Previously, the MEDAFI study reported a rate of 12%, who had chronic SCLs prior to ablation (16). Thus it may be difficult to determine whether the SCLs were procedure-related. Therefore, these findings should be interpreted with caution.

In the present study, the procedure-related complications were similar between CBA, HBA, LBA and RFA, and these were consistent with the previously published studies (36). However, occurrence of SCLs after CA for patients with AF remains a cogent cause of concern, as they may be associated with neurocognitive decline (6). Many previous studies have investigated the relationships between SCLs and neurocognitive outcomes. For example, the study by Vermeer et al. (8) and Sun et al. (37) showed that, SCLs led to an increased risk of dementia. However, as the study by Vermeer et al. (8) was almost 20 years ago, the risk relationship might have been changed due to the advances in treatment regimens and better control of risk factors (6). For example, the MEDAFI-trial found that, the incidence of SCLs was 8%, but no neurological effects occurred (16). Similarly, even in the MACPAF study, which found post-procedural SCLs in over 40% of patients, no abnormal result of neuropsychiatric assessment was detected (38). The most recent AXAFA-AFNET 5 trial also found similar cognitive function between those with and without SCLs (39).

These studies also demonstrated that, the majority of acute SCLs (up to 94%) were relatively small, and may resolve spontaneously within a short period of time (40). In addition, SCLs were also found in various cardiovascular interventions, including coronary angiography, valve surgery and coronary artery bypass grafting (41, 42). Therefore, SCLs may occur in various diagnostic and interventional cardiac catheterizations procedures, and the incidence and characteristics of SCLs may differ between different CA techniques, although no significant SCL-related cognitive adverse consequences were reported according to the recent evidence.

However, prevention is better than cure. It is crucial that electrophysiologists have an awareness of the factors that increase the risk of SCLs formation and the corresponding techniques for minimizing this risk (6). As mentioned above, the incidence of SCLs may be related to many other factors, such as pre-procedural anticoagulation, mean ACT during the procedure, exchange of catheters, and electrical cardioversion during the procedure. Intraprocedural ACT monitoring is recommended for AF ablation (6). Notably, an ACT level above 300 s during the procedure was associated with a reduced risk of left atrial thrombus detected by intracardiac echocardiography compared with 250–300 s (43). However, in the study using multivariate regression analysis by Wissner et al. (20), an ACT level over 250 s was not a predictor of SCLs on post-procedural MRI. In the present study, the mean ACT during the procedure was 296.4 s for CBA, 349.0 s for HBA, 262.0 s for LBA and 284.5 s for RFA. The mean ACT was comparable between CBA, HBA, LBA and RFA according to NMA and PMA, the relatively longer ACT time during CBA may contribute to its lower risk of SCLs and the highest rank to some extent.

Pre-procedural anticoagulation also seems to be an important factor in reducing the risk of SCLs (44). Increasing evidence has showed that continued oral anticoagulation leads to lower incidence of SCLs (22), and the protocol of anticoagulation (rivaroxaban) on the day of the ablation procedure was shown to reduce the incidence of SCLs compared with 24 h-pre-ablation bridging with heparin strategy (22). However, in the present study, it was not possible to compare uninterrupted with discontinued oral anticoagulant strategies due to lack of sufficient evidence. In addition, electrical cardioversion during CA procedure was also reported to increase the risk of SCLs (45). However, results remain controversial, as the study by Gaita et al. (46) found that pharmacologic or electrical cardioversion during the procedure could increase the risk of SCLs to 26%, compared with 9% in the patients who remained in sinus rhythm. In contrast, the study by Wissner et al. (20) failed to detect a significant relationship between SCLs and electrical cardioversion. In the most recent study that investigated the predicting factors of post-procedural SCLs using univariate and multivariate analyses, several patient-related characteristics, such as age and CHA2DS2-VASc score were implicated, whereas, the oral anticoagulant strategy, mean ACT after heparin injection, electrical cardioversion during the procedure and total procedure time were shown not to be positive risk factors (18).

Thus, based on the evidence of the present study, SCLs may occur after CA for patients with AF, and may be related to the different ablation techniques used. Though many risk factors for SCLs have been investigated, no consensus has been reached to date. Importantly, a universally accepted definition for SCLs and standard cerebral MRI diagnostic criteria are needed (7).

There are several limitations in this study. First, the total number of studies and the sample size included were relatively small; particularly, large-scale clinical trials were rare, which might influence the reliability of our results. Second, the analyses included both PAF and PerAF patients, and also mixed OAC protocols; however, further subgroup analysis could not be performed due to the lack of sufficient data. Third, considerable heterogeneities were detected when analyzing the outcomes such as procedure time; although further sensitive analyses were conducted, the interpretation of these results should still be taken with caution. In addition, the long time interval among the included studies might lead to certain bias, considering that the anticoagulation regimens have undergone changes. Finally, the protocols of the pre- and post-procedure cerebral MRI examinations varied across studies, which may lead to some bias when diagnosing the new SCLs. Nevertheless, our study is the first to provide a comprehensive analysis of SCL occurrence after different balloon-based ablation and RFA techniques for AF, with a scrutiny of all available trials. The results of the present study may provide new evidence, but further multicenter RCTs are still needed to confirm the findings.

## 6 Conclusions

CBA treatment was associated with a lower incidence of SCLs on MRI after AF procedure than LBA. Similar post-procedural SCL incidence was found between other ablation techniques, with CBA ranking highest. Total procedure-related complications were also similar between CBA, HBA, LBA and RFA. CBA needed shorter procedure time than LBA. Potential confounders like the scarcity of trials, a heterogeneous AF population, diverse OAC protocols, a lengthy time interval between studies, and varied MRI examination protocols could have affected the observed outcomes to some extent. Thus, further large-scale studies are still needed to improve the robustness of the conclusions.

## Data Availability

The original contributions presented in the study are included in the article/Supplementary Material, further inquiries can be directed to the corresponding authors.

## Supplementary material

Supplementary Figure S1 Funnel plot for the studies included based on the primary outcome OR = odds ratio.

Supplementary Figure S2 Forest plot for the outcome of total SCLs number from pairwise meta-analysis LBA = laser balloon ablation; CBA = cryoballoon ablation; RFA = radiofrequency ablation; OR = odds ratio.

Supplementary Figure S3 Forest plot for the outcome of procedure-related complications from pairwise meta-analysis HBA = hot balloon ablation; LBA = laser balloon ablation; CBA = cryoballoon ablation; RFA = radiofrequency ablation; OR = odds ratio.

Supplementary Figure S4 Forest plot for the outcome of procedure time from pairwise meta-analysis HBA = hot balloon ablation; LBA = laser balloon ablation; CBA = cryoballoon ablation; RFA = radiofrequency ablation; WMD = weighted mean difference.

Supplementary Figure S5 Forest plot for the outcome of mean ACT during procedure from pairwise meta-analysis HBA = hot balloon ablation; LBA = laser balloon ablation; CBA = cryoballoon ablation; RFA = radiofrequency ablation; WMD = weighted mean difference.
