# Supplementary material for: Silent cerebral lesions after catheter ablation for atrial fibrillation using cryoballoon, hotballoon, laserballoon and radiofrequency catheters: a Bayesian network meta-analysis
Source: Front Cardiovasc Med. 2025 Jan 14;11:1510468. doi: 10.3389/fcvm.2024.1510468 (PMC11772368; doi:10.3389/fcvm.2024.1510468)
Supplement: Supplementary file 1 [file Table1.docx]

**Supplementary Table** **S1** Estimated Differences of SCLs occurrences after different catheter ablation treatments for AF

| Treatment | CBA | HBA | RFA | LBA |
| --- | --- | --- | --- | --- |
| CBA | SCURA=81.1% | 0.9 (0.4-2.0) | 0.7 (0.4-1.2) | 0.6 (0.3-1.3) |
| HBA | - | SCURA=61.4% | 0.8 (0.3-1.8) | 0.7 (0.3-1.8) |
| RFA | - | - | SCURA=35.1% | 0.9 (0.4-1.9) |
| LBA | - | - | - | SCURA=22.4% |

Results are expressed as OR with 95% CrI in parentheses. Interventions are ordered according to SUCRA. The OR <1 indicates lower risk and the intervention listed in the left column is more beneficial than the one in the top row. CBA= cryoballoon ablation, HBA = hot balloon ablation, RFA = radiofrequency ablation, LBA = laser balloon ablation, SUCRA = surface under the cumulative ranking curve.
