# Supplementary figures and images for: Silent cerebral lesions after catheter ablation for atrial fibrillation using cryoballoon, hotballoon, laserballoon and radiofrequency catheters: a Bayesian network meta-analysis
Source: Front Cardiovasc Med. 2025 Jan 14;11:1510468. doi: 10.3389/fcvm.2024.1510468 (PMC11772368; doi:10.3389/fcvm.2024.1510468)

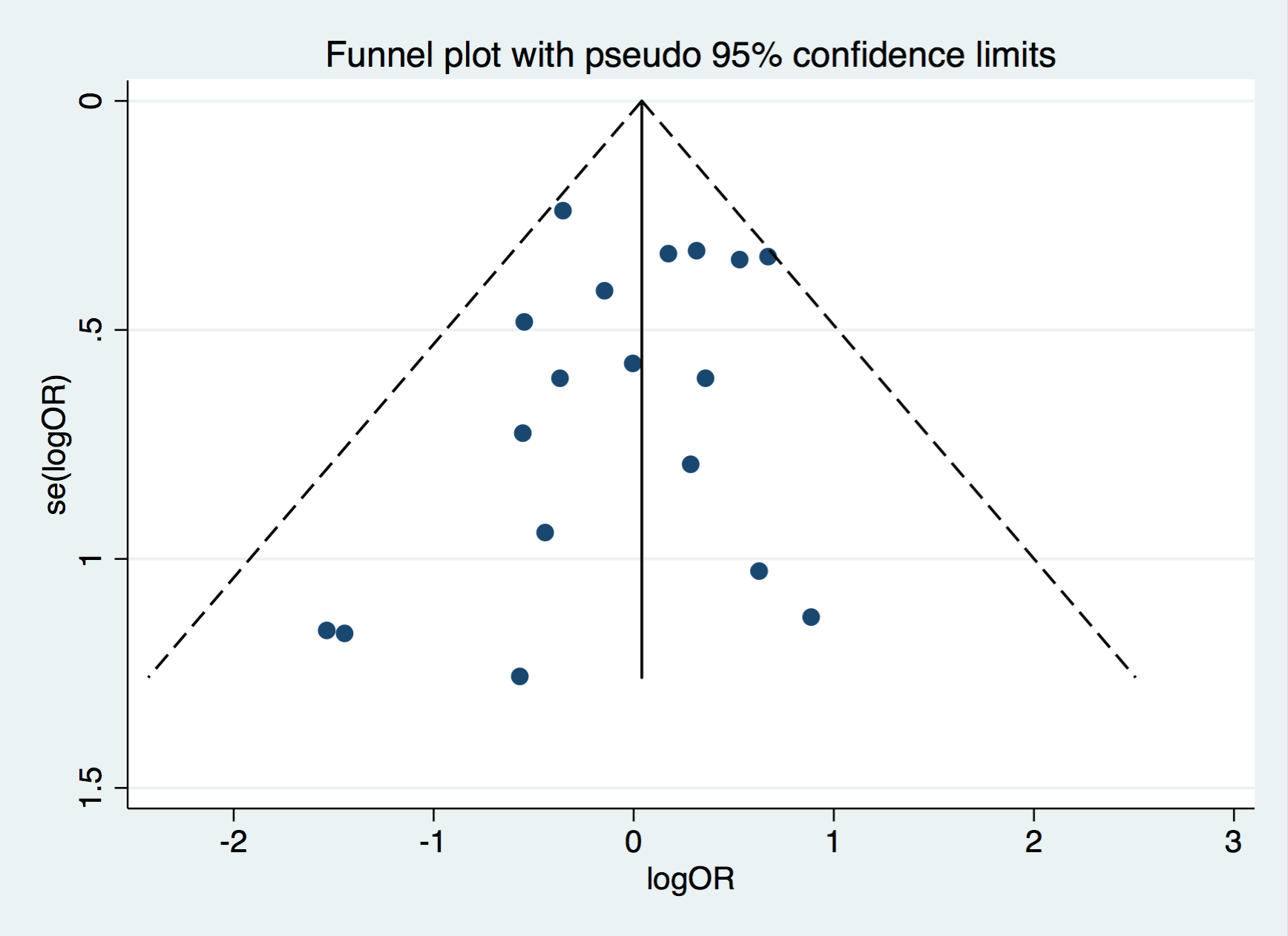

Supplement: Supplementary Figure S1 — Funnel plot for the studies included based on the primary outcome OR = odds ratio. [file Image1.tif]

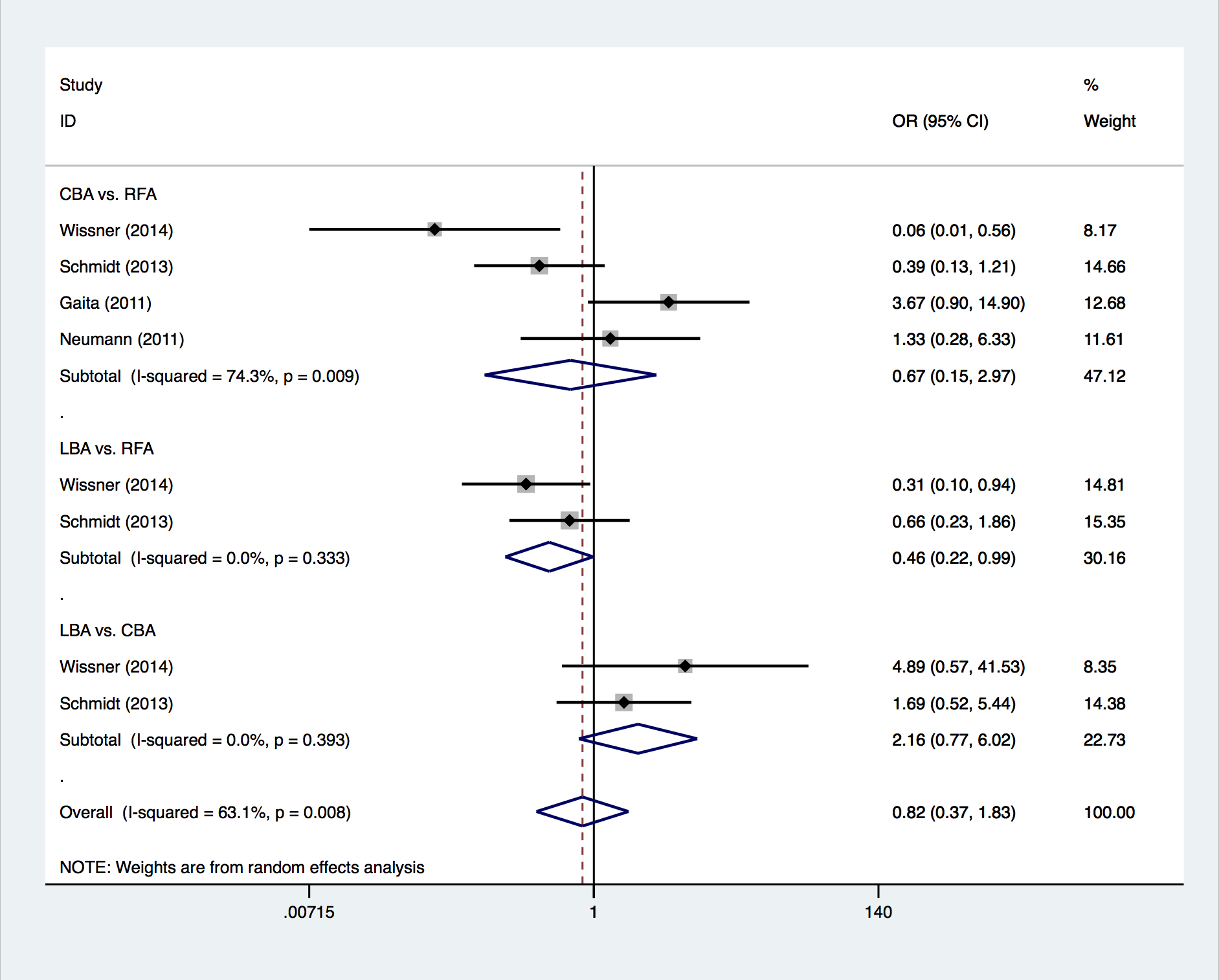

Supplement: Supplementary Figure S2 — Forest plot for the outcome of total SCLs number from pairwise meta-analysis LBA = laser balloon ablation; CBA = cryoballoon ablation; RFA = radiofrequency ablation; OR = odds ratio. [file Image2.tif]

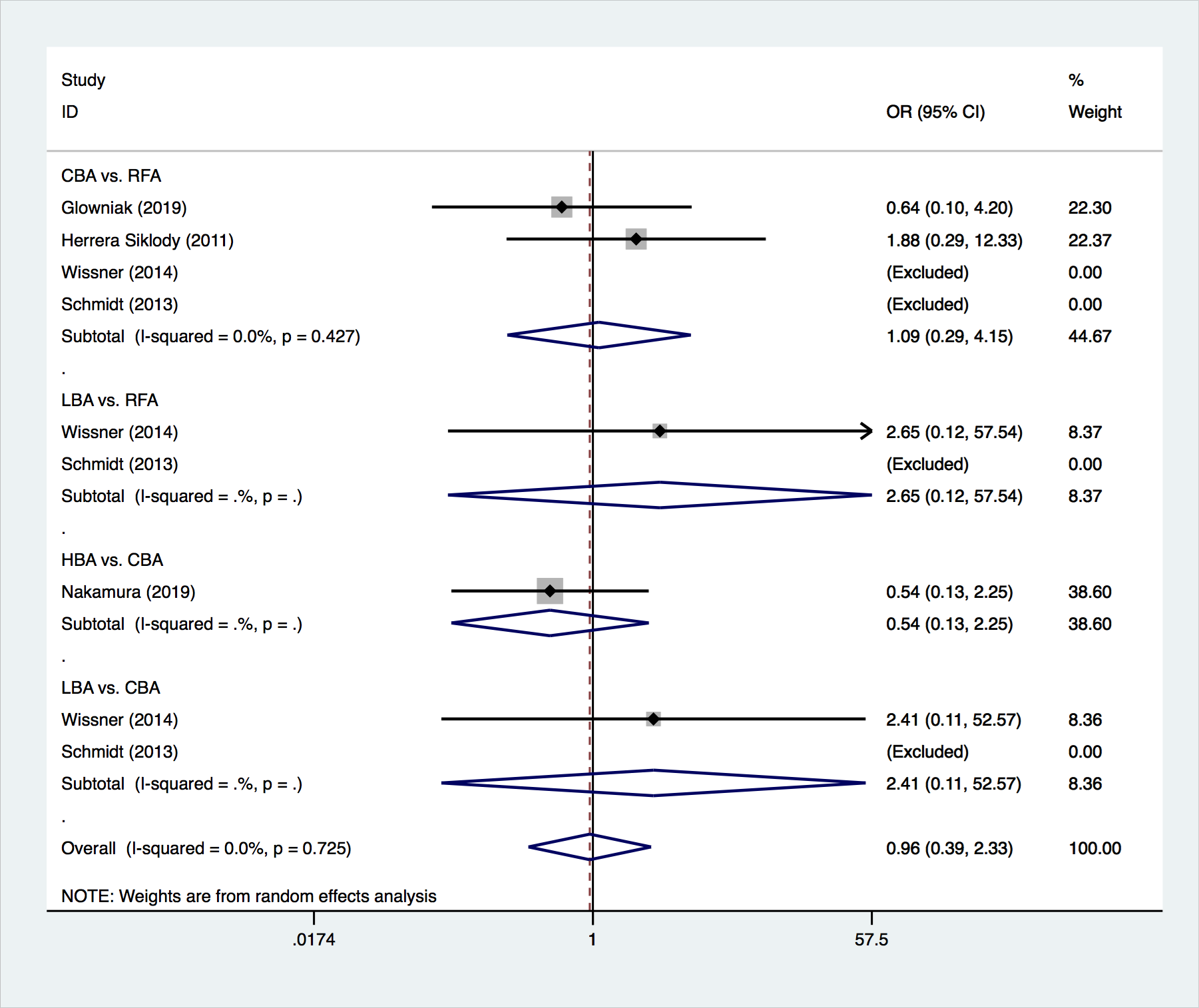

Supplement: Supplementary Figure S3 — Forest plot for the outcome of procedure-related complications from pairwise meta-analysis HBA = hot balloon ablation; LBA = laser balloon ablation; CBA = cryoballoon ablation; RFA = radiofrequency ablation; OR = odds ratio. [file Image3.tif]

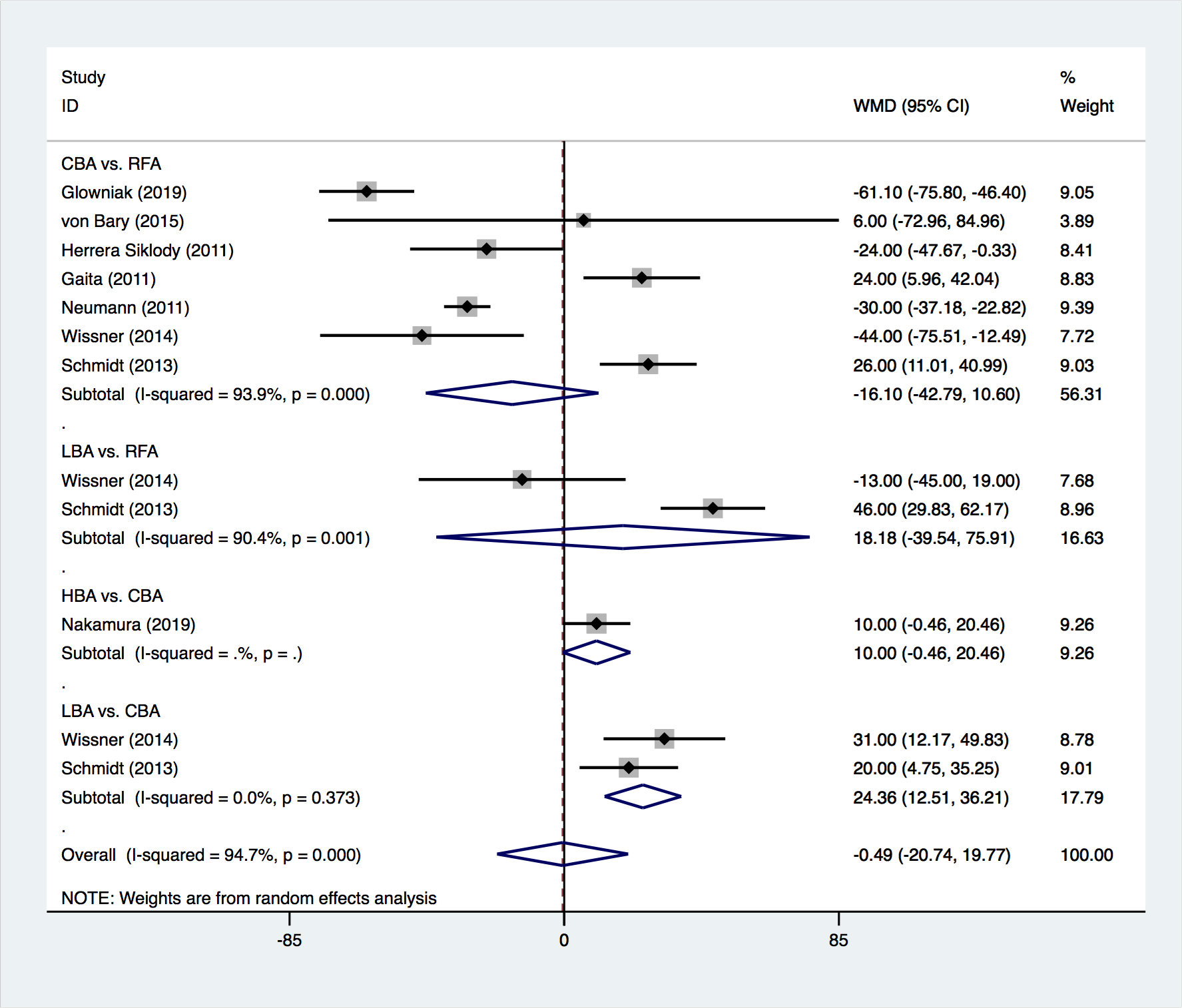

Supplement: Supplementary Figure S4 — Forest plot for the outcome of procedure time from pairwise meta-analysis HBA = hot balloon ablation; LBA = laser balloon ablation; CBA = cryoballoon ablation; RFA = radiofrequency ablation; WMD = weighted mean difference. [file Image4.tif]

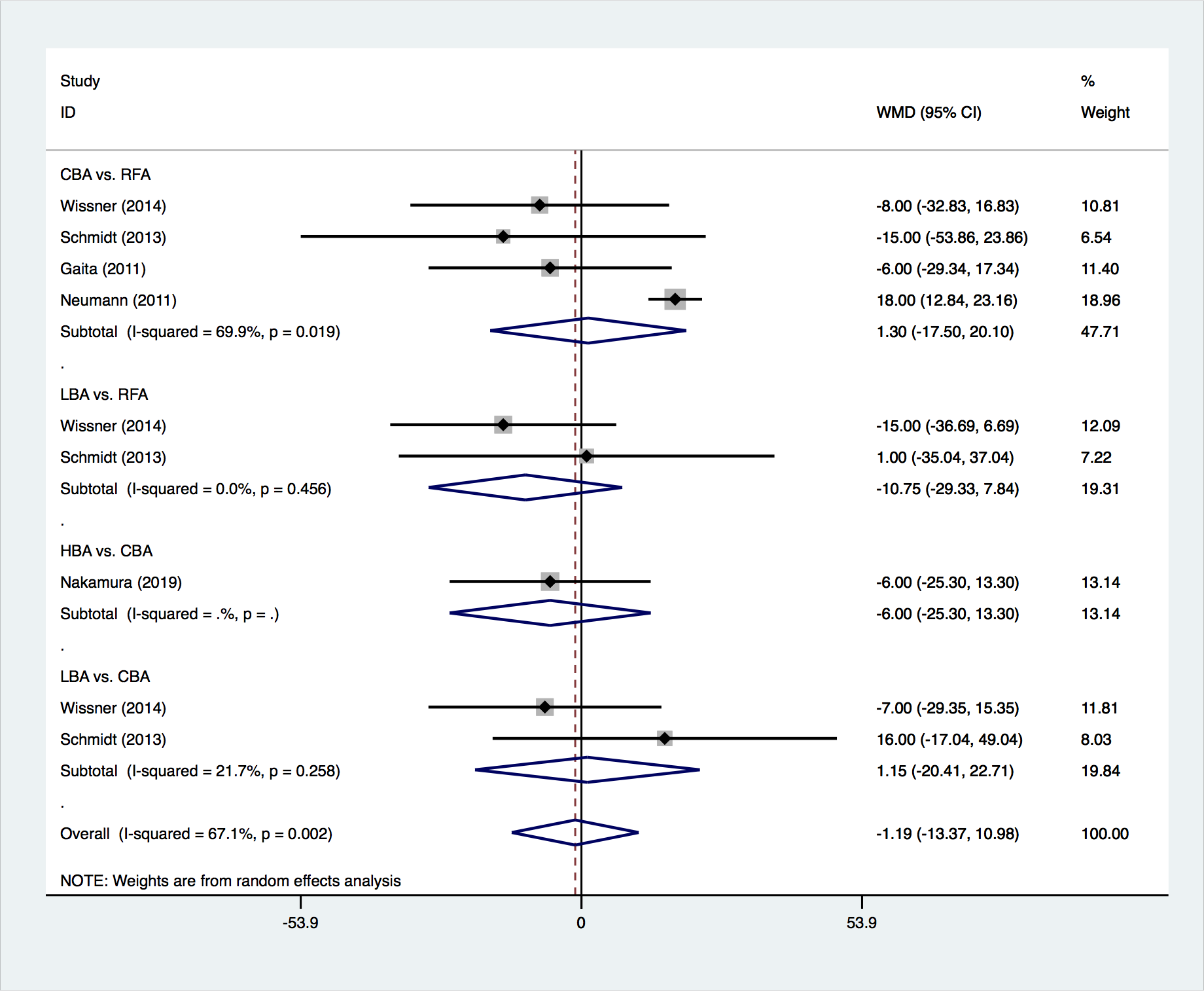

Supplement: Supplementary Figure S5 — Forest plot for the outcome of mean ACT during procedure from pairwise meta-analysis HBA = hot balloon ablation; LBA = laser balloon ablation; CBA = cryoballoon ablation; RFA = radiofrequency ablation; WMD = weighted mean difference. [file Image5.tif]
